# In the Period of Expectation

**Published:** 1890-11

**Authors:** W. E. Gladstone


### IN THE PERIOD OF EXPECTATION.

BY RT. HON. W. E. GLADSTONE.

When the foot of the Greek first, and afterward of the Roman, trod the streets of Jerusalem, when the treasures of the Hebrew books were unlocked to the Gentile world through the Septuagint, then there happened, we may justly assume, one of two things. There was, as we know upon strong heathen testimony, before the advent of our Lord, a universal and traditional expectation in the East that a great power was to arise in Judea and to subdue the world. How came it that so remarkable a conception, foreign to the cultivated communities of the Greek and the Italian peninsulas, and apparently menacing the continuance of the Roman dominion, should have been prevalent in the East?

The East had, indeed, at certain epochs supposed itself entitled to the mastery of the world ; hence, the wild expedition of Darius into Scythia and the repeated conflicts of Persia with the Greeks. It is not strange that this heritage should be reclaimed, for ideas of this kind are tenacious of life and easy of revival. But what is at first sight so strange is the choice of the spot from which deliverance was to proceed. It was not from any of the seats of ancient power, the fame of which was still on record, but from among the small, isolated, and undistinguished people who inhabited Palestine, and whose brief appearance on the stage of human affairs as conquerors in the time of King David was so slight in limit and in duration as to have inscribed no mark upon the page of general history. It had passed away, like the old empire of the Hittites ; they were also a people whose manners and institutions repelled rather than attracted the sympathy of the world.

One supposition explanatory of this remarkable expectation might be that it had lived on from prehistoric times in feebleness and obscurity, but had come to the front when the East felt pressing on it from Rome the hard hand of power, welding it for the first time by a permanent system into uniformity of servitude or inferiority, from which it panted for deliverance. But it seems more probable that the Jewish Scriptures, which had for two centuries become known by translation into Greek, were themselves the fountain head of this most remarkable anticipation ; and in that case it probably proceeded in an eminent degree from the Messianic Psalms, which were of all the available evidence the part most in the eye and mind of the people.

